# Psychological Well-Being among Adolescents and Youth

**DOI:** 10.3390/healthcare10102059

**Published:** 2022-10-17

**Authors:** Costanza Scaffidi Abbate, Silvana Miceli

**Affiliations:** Department of Psychology, Educational Science and Human Movement, University of Palermo, 90128 Palermo, Italy

Psychology has long conceived of individuals in terms of psychopathology and dysfunction. It has been concerned with repairing damage according to a disease model of human functioning, focusing little on constructing positive qualities [1]. Adolescence has been no exception and is often seen as a period filled with problems and difficulties [2], collecting a wealth of data on risk factors, problematic behaviors, and prevention formulas [3,4].

With the popularity of positive psychology, there has been an increasing number of studies focusing on the positive effects of the well-being on individuals and exploring the general and situation-specific factors that influence individual well-being in different cultural and social contexts [5,6,7].

Today, the construct of well-being has, at last, assumed increasing importance in various areas of psychology by identifying several factors of personal order (personality dispositions, self-esteem, perception of control), interpersonal (social support), and socioeconomic (income, level of education), variables that can influence well-being to a greater or lesser extent. Indeed, some problems arise when providing a shared definition of well-being or identifying a comprehensive theory [8]. One limitation is the very nature of the construct, which has been investigated with different tools that have highlighted aspects and dimensions that are not always overlapping, which poorly explain the relationships between the variables. Ryan and Deci [9] divided studies on well-being into two major strands, the first termed “hedonic” and the second “eudaimonic”. The first contemplates a hierarchical structure at the apex of which is the concept of well-being itself and, at lower levels, subcomponents, such as satisfaction with one’s activities or positive and negative effects [10,11]; the second model emphasizes the multidimensionality of the concept of well-being, the basic principles of which are the concepts of happiness and pleasure, and the various dimensions are to follow Ryff’s [12] model, autonomy, fulfillment, and the different personal domains. Both perspectives refer to an idea of well-being understood not as merely the absence of illness but as self-realization and optimal functioning of the optimal mind, placing themselves within that strand of so-called positive psychology, which emphasizes the concepts of happiness, positive emotion, and psychological well-being [1] and whose goal is to understand how to promote the individual and societal level, the health of the individual improves their quality of life [13] and implement the dissemination of a culture of well-being.

This paradigmatic shift has also been reflected in adolescent studies in the last two decades [14]. The image has been abandoned as a developmental stage characterized “physiologically” by deep existential distress and severe disequilibrium. From a current perspective, adolescence represents a critical phase of the life cycle during which the young person is grappling with complex developmental tasks, such as the reorganization and structuring of the concept of self and the completion of the individuation-separation process. At the same time, adolescence is neither the only, nor the most problematic phase of the life cycle of life. Most importantly, it does not represent a period dominated by negative emotions for most adolescents. Most adolescents tend to provide a positive self-image [15] and can be able to cope with the challenges and developmental tasks characteristic of this stage of life [16]; the very concept of adolescent crisis can be reconsidered as an opportunity that the adolescent has to test themselves and from which they can emerge from it, fortified and with more significant resources. Thus, if adolescents can experience positive self-perception and show themselves capable of seizing the opportunities that life offers them, it is crucial to understand more deeply what can foster greater or lesser levels of personal well-being [17].

Understanding what risk and protective factors can foster or undermine the well-being of young people is a challenge to which many disciplines, from psychology to psychiatry and sociology, are making valuable contributions. Studies from different fields that primarily adopt a multifactorial perspective, capable of considering both individual characteristics and the characteristics of the contexts in which the adolescent lives and their relationships, as well as the interaction between risk and protective factors, are welcome in this Special Issue entitled “Psychological Well-Being for Adolescents and Youths”.

## Data Availability

Not applicable.
